# *De Novo* Assembly and Annotation of the Larval Transcriptome of Two Spadefoot Toads Widely Divergent in Developmental Rate

**DOI:** 10.1534/g3.119.400389

**Published:** 2019-06-19

**Authors:** H. Christoph Liedtke, Jèssica Gómez Garrido, Anna Esteve-Codina, Marta Gut, Tyler Alioto, Ivan Gomez-Mestre

**Affiliations:** * Ecology, Evolution and Development Group, Doñana Biological Station (CSIC), Seville 41092, Spain,; † CNAG‐CRG, Centre for Genomic Regulation (CRG), The Barcelona Institute of Science and Technology, Baldiri Reixac 4, Barcelona 08028, Spain, and; ‡ Universitat Pompeu Fabra (UPF), Barcelona, Spain

**Keywords:** Amphibian development, developmental rate, amphibian transcriptomes, developmental plasticity, cross-species comparisons

## Abstract

Amphibians are highly vulnerable and diverse vertebrates for which we still have modest genomic resources. Amphibian larvae are key components of continental wetlands, where they have strong influences on energy fluxes, nutrient cycling, and community structure. Amphibian larvae are highly sensitive to environmental conditions and can often alter their physiology, behavior and even morphology in response to the local conditions experienced, although we still know relatively little about the transcriptomic changes that enable such plasticity. Here we contribute the larval transcriptomes of two spadefoot toad species with divergent developmental rates and degree of developmental plasticity in response to pond drying.

Most amphibian species exhibit a complex life-cycle including two or more life stages separated by an ontogenetic switch point such as hatching or metamorphosis. Adaptations to divergent environments can require the modification of the timing of such switch points and the relative investment in growth and differentiation between subsequent stages (Moran 1994). Such alterations of developmental trajectories, however, often have substantial repercussions at several organismal levels, from physiology to morphology and even genomic structure. Adaptive divergence in developmental rate tracking aquatic habitats of different duration in spadefoot toads is a well-known example of this. Spadefoot toads from Europe and northern Africa typically have tadpoles that grow to be quite large over a long larval period, but can otherwise accelerate development and precipitate metamorphosis if at risk of pond drying, whereas north American spadefoot toads tend to have smaller tadpoles that develop faster and are less capable of further developmental acceleration (Buchholz and Hayes 2002). At opposite ends of this spectrum, we find *Pelobates cultripes*, distributed throughout most of the Iberian Peninsula and southern France, and *Scaphiopus couchii*, distributed across southwestern USA to northern Mexico. *Pelobates cultripes* larvae grow quite large (> 16 g) and can take up to 6 months to reach metamorphosis, whereas *S. couchii* tadpoles are much smaller (1.5-2 g) and can develop to metamorphosis in as little as eight days. Such developmental acceleration is rather energetically demanding and requires a substantial increase in metabolic activity (Kulkarni *et al.* 2017), hence causing oxidative stress (Gomez-Mestre *et al.* 2013). Precipitating metamorphosis alters growth and developmental trajectories, and as these are non-isometrical for different parts of the body, the emerging metamorphs are not only smaller but also have relatively shorter limbs (Gomez-Mestre *et al.* 2010; Kulkarni *et al.* 2011; Johansson and Richter-Boix 2013). Developmental acceleration is achieved through neuroendocrine regulation mainly resulting in increased corticosterone and thyroid hormone levels (Denver 2009; Kulkarni *et al.* 2017), as well as through differential expression of hormone receptors (Gomez-Mestre *et al.* 2013). Interestingly, the canalized fast development of *S. couchii* to a large extent mirrors the environmentally-induced accelerated state of the more plastic *P. cultripes* (Kulkarni *et al.* 2017).

At the genomic level, evolutionary divergences in developmental rate seem to leave a big imprint on whole genomes with some studies showing that fast developmental rates are often associated with smaller genome sizes (Wyngaard *et al.* 2005; Alfsnes *et al.* 2017). The rule also holds true for amphibians, whether at a large macroevolutionary scale (Liedtke *et al.* 2018) or focused on specific species groups (Jockusch 1997). Spadefoot toads present broad differences in developmental rate across species, which are consequently also reflected in large differences in genome size (Zeng *et al.* 2014): slow developing *P. cultripes* has a large genome (∼3.9 Gbp), whereas fast developing *S. couchii* has only about one third its size (∼1.5 Gbp). Here we present a first description of the transcriptomes of these species at the onset of metamorphosis to explore the potential consequences of such dramatic divergence in their genomes and to uncover the transcriptomic basis of their differences in developmental rate.

The NCBI Transcriptome Shotgun assemblies database currently lists transcriptome assemblies for 26 species of amphibians and of those, only four are larval phase transcriptomes: *Rhinella marina* (Richardson *et al.* 2018), *Microhyla fissipes* (Zhao *et al.* 2016), *Rana* (*Lithobates*) *catesbeiana* and *Xenopus laevis* (Birol *et al.* 2015). The addition of transcriptomes for the larval phases of two more species, especially as they represent a distinct evolutionary lineage, is therefore a significant contribution to the current knowledgebase.

## Methods

### Sample collection, total RNA extraction and sequencing

The experimental procedures described below were approved by the CSIC IACUC committee (permit #17_01 CEEA-EBD). Three egg clutches of *P. cultripes* were collected from a natural pond in Doñana National Park, southwestern Spain, brought to a walk-in chamber in the laboratories of Doñana Biological Station (EBD-CSIC) and placed in a plastic tray with carbon-filtered dechlorinated tap water with aerators to ensure adequate oxygenation. Three clutches of *S. couchii* were spawned in the laboratory at EBD-CSIC by adult pairs obtained from a captive colony at Daniel Buchholz’s laboratory at the University of Cincinnati (USA), and originally collected in SE Arizona. Adults were hormonally stimulated to breed by intraperitoneally injecting 20–100 μL of 1 μg/100 μL GnRH agonist (des-Gly, [D-His(Bzl)]-luteinizing hormone releasing hormone ethylamide, Sigma). Upon hatching, we transferred tadpoles from each clutch of each species to 3 L plastic containers with dechlorinated tap water where they were individually kept under standard conditions of 24°, 12:12 L:D photoperiod, and *ad libitum* food supply consisting of finely powdered rabbit chow. Tadpoles were stage-matched so that when they advanced in development to Gosner stage 35, as determined by the individualization of all five toes (Gosner 1960), we euthanized twelve individuals per species via MS-222 overdose, eviscerated them to avoid interferences from fecal material, and snap-froze them in liquid nitrogen. We extracted whole-body total RNA from each tadpole using Trizol reagent following the manufacturer’s protocol (Invitrogen). Total RNA was assayed for quantity and quality using Qubit RNA HS Assay (Life Technologies) and RNA 6000 Nano Assay on a Bioanalyzer 2100 (Agilent).

The RNASeq libraries were prepared from total RNA using the TruSeqStranded mRNA LT Sample Prep Kit (Illumina Inc., Rev.E, October 2013). Briefly, 500ng of total RNA was used as the input material and was enriched for the mRNA fraction using oligo-dT magnetic beads. The mRNA was fragmented in the presence of divalent metal cations. The second strand cDNA synthesis was performed in the presence of dUTP instead of dTTP, this allowed to achieve the strand specificity. The blunt-ended double stranded cDNA was 3′adenylated and Illumina indexed adapters were ligated. The ligation product was enriched with 15 PCR cycles and the final library was validated on an Agilent 2100 Bioanalyzer with the DNA 7500 assay.

Each library was sequenced using TruSeq SBS Kit v3-HS, in paired end mode with the read length 2x76bp. We generated on average 38 million paired-end reads for each sample in a fraction of a sequencing lane on HiSeq2000 (Illumina) following the manufacturer’s protocol. Images analysis, base calling and quality scoring of the run were processed using the manufacturer’s software Real Time Analysis (RTA 1.13.48) and followed by generation of FASTQ sequence files by CASAVA 1.8.

### Assembling de novo transcriptomes of P. cultripes and S. couchii

Quality of raw reads was inspected using FASTQC (https://www.bioinformatics.babraham.ac.uk/projects/fastqc/) and MULTIQC (Ewels *et al.* 2016). Assembly was performed using Trinity v2.4.0 (Haas *et al.* 2013) for the two species separately. Reads from all samples per species were combined, trimmed (using default Trimmomatic settings SLIDINGWINDOW:4:5 LEADING:5 TRAILING:5 MINLEN:25)(Bolger *et al.* 2014) and normalized using *in silico* normalization with default Trinity settings (flags used:–trimmomatic–normalize_max_read_cov 50).

### Assessment of transcriptome quality and completeness

Transcriptome quality in terms of read representation was evaluated by mapping the normalized reads (pairs only) back onto the transcriptome using Bowtie2 v2.3.2 (Langmead and Salzberg 2012). Completeness in terms of gene content was assessed using BUSCO v3.0.2 (Simão *et al.* 2015) with the tetrapoda-odb9 database as a reference as well as by running blastx (E-value cut off E ≤ 1e^-20^) against both the SwissProt database (downloaded on 01.11.2017) and the *Xenopus tropicalis* proteome (Ensemble JGI 4.2; downloaded on 03.11.2017) with a stringent Evalue criteria of ≤ 1e^-20^. The count of full-length transcripts with blastx hits was based on grouped high scoring segment pairs per transcript to avoid multiple fragments per transcript aligning to a single protein sequence.

### Functional annotation

We used Trinotate v3.0 (https://trinotate.github.io/) to annotate the transcriptome. This involves finding similarities to known proteins by querying transcripts against the Swissprot database (accessed in June 2018) (The UniProt Consortium 2017) (blastx with a cut-off of E-value ≤ 1e^-5^). Moreover, likely coding regions were detected with TransDecoder (https://github.com/TransDecoder) and resulting protein products (coding sequence; CDS) were matched against both the complete Swissprot database and a subset including only vertebrate genes, using blastp (E-value ≤ 1e^-5^), and a conserved protein domain search was conducted using HMMER (http://hmmer.org/) on the Pfam database (Finn *et al.* 2016). SignalP v4.1 (Petersen *et al.* 2011) and TmHMM v2.0 (http://www.cbs.dtu.dk/services/TMHMM/) were used to predict signal peptides and transmembrane regions respectively. Finally, gene ontology identifiers were assigned to transcripts based on available annotations from best-matching Swissprot entries. Trinotate also provides KEGG (Kyoto Encycopedia of Genes and Genomes; http://www.genome.jp/kegg/) and EggNOG (Huerta-Cepas *et al.* 2016) annotations. Exploring the Trinotate output was facilitated using the TrinotateR R package (https://github.com/cstubben/trinotateR).

### Expression-based filtering and cross-species comparisons

Transcripts with low expression levels have questionable biological relevance. Because of this, abundances were estimated using Kallisto v0.4.3.1 (Bray *et al.* 2016), and the transcriptome was filtered to retain only transcripts with more than 1 Transcript Per Million (TPM). This threshold roughly coincides with a notable influx of lowly expressed transcripts when plotting the number of transcripts as a function of minimum TPM thresholds. In addition, any transcripts flagged by NCBI as containing potential vector, adaptor or primer contaminations were also filtered out.

The PANTHER classification scheme (Mi *et al.* 2013) for *Xenopus tropicalis* was used to organize gene function and ontology. The filtered transcriptomes were annotated using the *Xenopus* Ensembl protein identifiers recovered from the blastx search (see above) and using the PANTHER web server, we performed both functional classifications and a statistical overrepresentation test (Fisher’s exact test with a false discovery rate correction) to investigate which genes are significantly (*P* < 0.05) over or under represented in the filtered transcriptomes compared to the *X. tropicalis* reference. Further functional classification and gene pathway enrichment was performed using gProfiler (g:GOST; Raudvere *et al.* 2019) using the accompanying R package gprofiler2 v0.1.3. The expression-filtered *Xenopus* protein identifiers were queried against the Gene Ontology – Biological Process, KEGG and Reactome databases (Fabregat *et al.* 2017). An overrepresentation test was performed to calculate the probability that the intersection of query and a functional category has arisen by chance, using *X. tropicalis* as the reference organism, limiting the gene domain to only annotated genes and excluding GO electronic annotations. The gene list was ranked in order of decreasing expression (using the mean TPM per gene across all biological replicates) and treated as an ordered query to perform an incremental enrichment analysis, giving more importance to higher expressed terms. P-values were adjusted for multiple testing using the g:SCS algorithm. Functional groupings and gene networks of significantly overrepresented/enriched terms and pathways were further explored using the EnrichmentMap add-on in Cytoscape v3.7.1 (Shannon *et al.* 2003; Merico *et al.* 2010).

Finally, OrthoFinder v2.2.3 (Emms and Kelly 2015) was used to find orthologous genes across the two species. OrthoFinder was run with default settings, taking the TransDecoder predicted CDS of both *P. cultripes* and *S. couchii* as the input, as well as the proteome of *X. tropicalis* to provide context (as an ‘outgroup’).

### Data availability

The data sets supporting the results of this article are available as supporting information. Specifically, we provide quality assessment results of both BUSCO and BowTie2, the unfiltered transcriptome assemblies plus their transdecoded CDS files and annotations in the form of Trinotate summary tables and Panther annotation lists. In addition, all raw reads as well as the expression filtered transcriptome assemblies are deposited on the NCBI’s Sequence Read Archive [SRA; SRP161446] and Transcriptome Shotgun Assembly database [TSA; *P. cultripes*: GHBH01000000; *S. couchii*: GHBO01000000], under BioProject [PRJNA490256]. Supplemental material available at FigShare: https://doi.org/10.25387/g3.8201825.

## Results and Discussion

### Transcriptome comparison and quality assessment

The twelve *P. cultripes* samples each consisted of 30.5-43.3 million, 101bp paired-end reads (888.3 million reads in total) pooling to 84.2 million post-normalization pair-end reads used for the assembly (10.5% of total). Trinity generated 753,223 transcript contigs with median length 362, of which 428,406 clustered into ‘genes’ (transcript clusters with shared sequence content; Table 1). Bowtie2 mapped 83.96% of the reads back onto the transcriptome (Supporting Data 1). In comparison, the *S. couchii* samples consisted of 32.1-53.9 million, 101bp reads (958.8 million reads in total) with 84.4 million post-normalization pair-end reads used for the final assembly (9.19%). 657,280 transcripts were generated by Trinity with a median length of 432bp clustering into 381,135 ‘genes’ (Table 1). Bowtie2 mapped 90.71% of the reads back onto the transcriptome. The Trinity-assembled, unfiltered transcriptomes for both species are available as Supporting Data 2.

**Table 1:** Transcriptome assembly statistics for both tadpole species. Summaries for Trinity outputs are given both at the transcript and at the ‘gene’ level

	*P. cultripes*	*S. couchii*
*Total number of raw reads*	888,265,444	958,782,922
*Number of in silico normalized reads*	84,209,684	84,420,786
*Number of read pairs aligned to assembly*	419,274,288 (94.4%)	453,683,501 (94.6%)
*Number of proper pair reads aligned to assembly*	359,039,281 (85.6%)	425,833,848 (93.9%)
*N50 of transcripts | longest isoform per ‘gene’*	1,496bp | 731bp	2,057bp | 872bp
*Number of Trinity transcripts | ‘genes’*	753,223 | 428,406	657,280 | 381,135
*Size of transcript | longest isoform per ‘gene’:*		
*Total*	581,464,720bp | 237,111,496bp	644,907,581bp | 232,600,864bp
*Median*	362bp | 313bp	432bp | 331bp
*Average*	771.97bp | 553.47bp	981.18bp | 610.28bp

The BUSCO results support near-complete gene sequence information for 89.7% of genes in the *P. cultripes* transcriptome with only 7.4% of the genes being fragmented and 2.9% missing. The quality of the *S. couchii* assembly was similar with 86.6% complete sequence information, 10.5% fragmented genes and 2.9% missing (Supporting Data 3).

**Figure 1:**
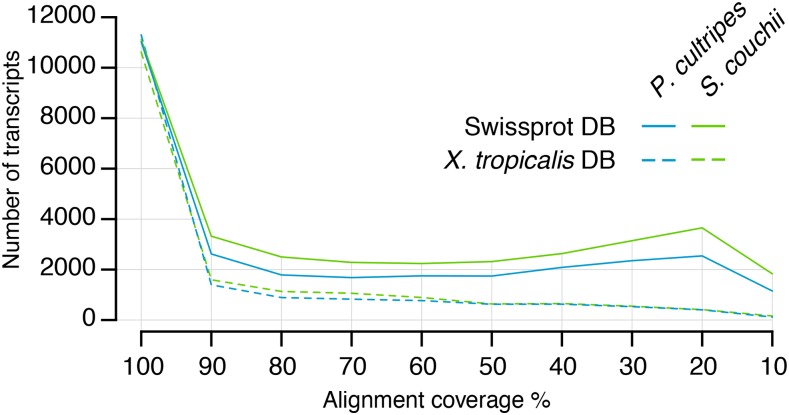
Number of transcripts (using grouped highest scoring segment pairs) per alignment coverage bins when querying against the SwissProt and *Xenpus tropicalis* proteome sequence databases.

Querying the Trinity assembly against both the Swissprot database and the *X. tropicalis* proteome (using blastx) revealed large numbers of fully reconstructed coding transcripts, with 13,645 Swissprot proteins and 12,715 *X. tropicalis* proteins represented by nearly full-length transcripts (>80% alignment coverage) in the *P. cultripes* assembly, and 14,429 Swissprot proteins and 12,216 *X. tropicalis* proteins in the *S. couchii* assembly (Figure 1; Supporting Data 4).

### Functional annotation

Gene annotation via the Trinotate pipeline is useful for providing biological context to the assembled transcriptomes (Trinotate tables available as Supporting Data 5). Querying (using blastx) the SwissProt database with the Trinity assembly allowed for the annotation of 162,031 *P. cultripes* and 204,646 *S. couchii* transcripts. Gene Ontology (GO) derived from these hits resulted in 18,585 unique (out of a total of 1,626,015) GO annotations for *P. cultripes* and 19,917 unique (out of a total 2,155,849) GO annotations for *S. couchii*.

TransDecoder recovered fewer candidate-coding regions for *P. cultripes* (154,906) than for *S. couchii* (175,331; Table 2). This corresponds to 36.2% and 46.1% of the Trinity-identified ‘genes’ for *P. cultripes* and *S. couchii*, respectively. Homology searches using blastp against the entire Swissprot database were able to annotate 108,881 and 132,578 of the trasdecoded CDS, and 107,199 and 130,847 when searching the vertebrates-only database (Table 2). Of the sequences with vertebrate gene hits, 24,327 and 27,309 unique vertebrate swissprot proteins were identified for *P. cultripes* and *S. couchii* (genes with unique UniProtKB-IDs). Of these, the two species share 56.5% (18,651 proteins), with 17.2% being unique to *P. cultripes* (5,676 proteins) and 26.2% unique to *S. couchii* (8,658 proteins). Similarly, the number of hits of candidate coding regions against other databases including pfam, signalP, tmHMM, KEGG and EggNOG was greater for *S. couchii* than *P. cultripes* (Table 2).

**Table 2:** Number of unique | total TransDecoder-predicted candidate genes with annotations via different search tools and databases (summary of Trinotate results)

	*P. cultripes*	*S. couchii*
*TransDecoder predicted coding regions (ORFs)*	154,906	175,331
*Protein hits (blastp - SwissProt)*	79,504 | 108,881	91,337 | 132,578
*Protein hits (blastp – SwissProt vertebrates only)*	77,924 | 107,199	89,704 | 130,847
*pfam hits (HMMER search)*	65,597 | 91,929	76,741 | 112,740
*signalP predicted peptides*	3,943 | 10,981	4,114 | 13,097
*tmHMM predicted transmembrane proteins*	17,990 | 25,833	19,881 | 29,991
*GO Pfam*	2,471 | 57,308	2,583 | 71,966
*KEGG*	31,313 | 127,707	40,765 | 174,712
*EggNOG*	8,125| 117,983	8,681| 156,494

### Expression-based filtering and cross-species comparisons

Expression-based filtering results in only 20.8% of the *P. cultripes* transcripts and 26.8% *S. couchii* transcripts to be retained. Post-NCBI screening, the transcriptomes consisted of 153,520 transcripts for *P. cultripes* and 175,261 for *S. couchii* corresponding to 42,949 and 60,662 transdecoded peptide sequences respectively. The number of predicted coding sequences post expression filtering is in the range of published amphibian larval transcriptomes [*R. catesbeiana*: 51,720 CDS (Birol *et al.* 2015), *M. fissipes* 51,506 CDS (Zhao *et al.* 2016), *R. marina* 62, 365 CDS (Richardson *et al.* 2018)].

**Figure 2:**
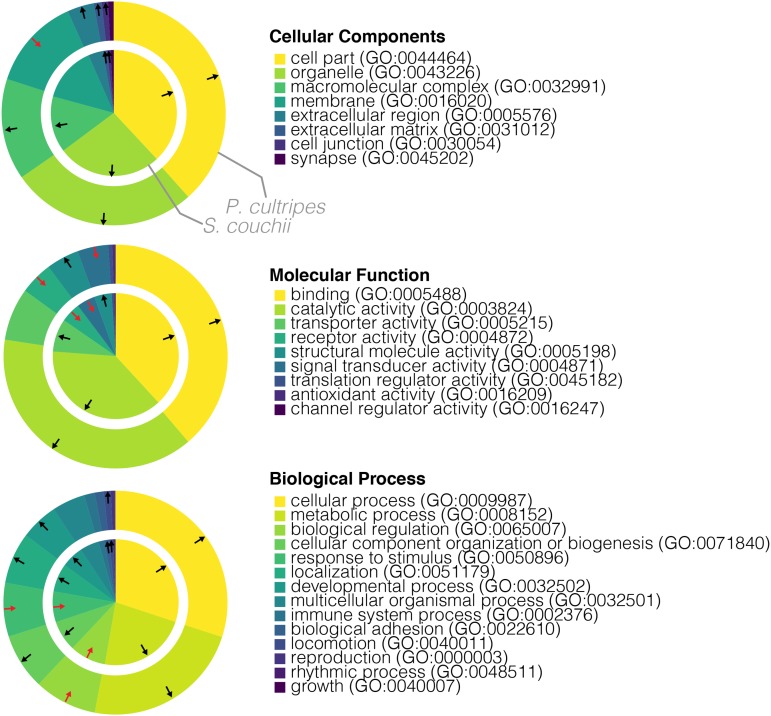
PANTHER functional classification of transcriptomes. Wedge size reflect number of unique genes per category and out/in, black/red arrow annotations specify significant over/under representation of the GO-slim term compared to the *X. tropicalis* reference database.

The PANTHER GO-slim classification system designed for *X. tropicalis* provides a curated, functional classification scheme of GO terms and allows for relative over or underrepresentation of terms to be assessed in relation to the reference (in this case *X. tropicalis*) database. For *P. cultripes*, 10,666 out of 13,421unique annotations could be mapped to PANTHER genes, and 11,598 out of 14,811 annotations for *S. couchii*. The relative abundances of GO terms, as well as their statistical representation in relation to *X. tropicalis*, are highly comparable across the two species (Figure 2). Cell parts and organelles (cellular components; CC), binding and catalytic activity (molecular function; MF) and cellular and metabolic processes (biological processes; BP) make up more than half of the terms for each ontology, in each case being significantly over represented in both species compared to in *X. tropicalis*. Underrepresented terms are less common, restricted only to genes related to receptor and signal transducer activity (MF) and biological regulation and response to stimulus (BP) in both species, as well as membrane genes (CC) in *P. cultripes* only. Bar charts showing the over and underrepresentation of each PANTHER term per species per ontology are provided as supporting data (Supporting Data 6).

**Figure 3:**
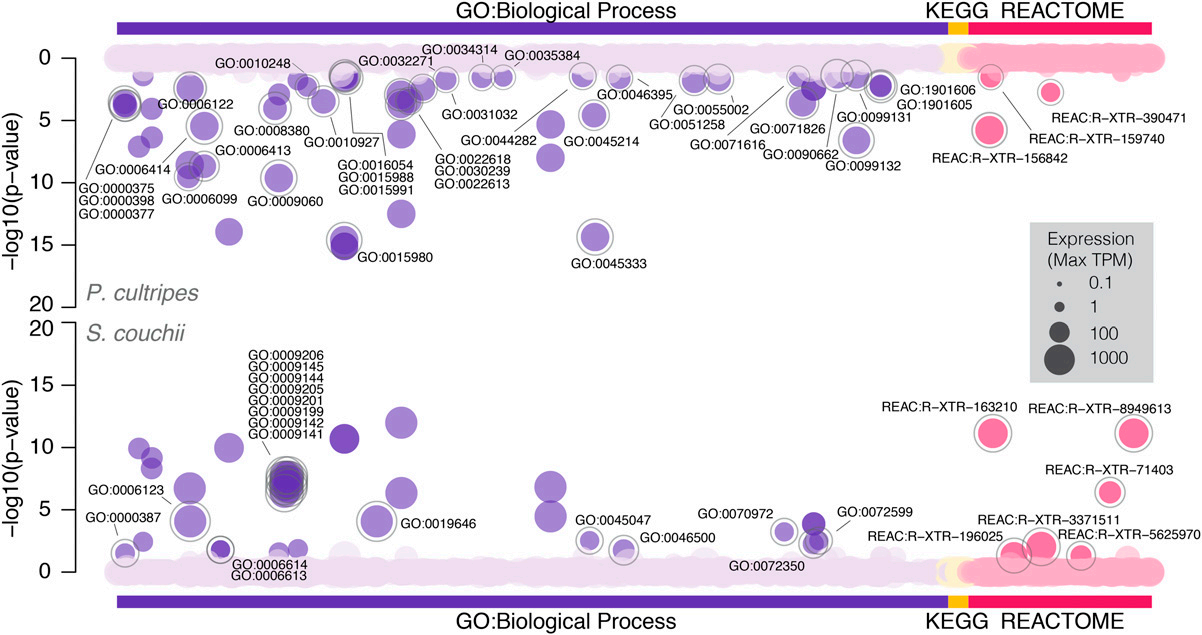
gProfiler gene pathway enrichment showing symmetrical Manhattan plots of annotated terms and gene pathways for *P. cultripes* (above) and *S. couchii* (below). Color code reflects annotation domains (Gene Ontology - Biological Processes, KEGG and Reactome gene pathways) with darker shading highlighting overrepresented terms (adj. *P* < 0.05). Term size reflects mean expression (TPM) and overrepresented terms that are unique to each species are outlined in gray and labeled.

gProfiler could map 12,391 genes out of a total query of 13,421 to 8,229 functional terms for *P. cultripes*, of which 54 are significantly enriched (adjusted p-value < 0.05). In comparison, for *S. couchii*, gProfiler could map 13,315 out of 14,811 queried genes to 8,455 functional terms, of which 40 are significantly enriched (Supporting Data 7). For both species, the majority of enriched terms were GO terms, with the addition of nine Reactome pathways (three for *P. cultripes* and six for *S. couchii*). No KEGG pathways were enriched for either species (Figure 3; Table 3). Sixty-two of the significantly enriched terms and pathways were unique to each species, with only 16 shared terms, all within the GO term domain space (Figure 3; Table 3). When exploring this subset of significant terms and pathways further using Cytoscape, the most extensively represented clusters include ribonucleoside triphosphate metabolic processes (both species, though primarily *S. couchii*), mitochondrial electron transport (both species) and cytoplasmic initiation complex (both species), with the most represented species-specific terms being actomyosin structure organization (*P. cultripes*) and protein targeting to endoplasmic reticulum (*S. couchii*; Supporting Data 8). Genes related to thyroid hormone regulated development or oxidative stress were not recovered as significantly overrepresented in either species. Interestingly, however, the enriched annotations for *S. couchii* showed a notable representation of nucleoside triphosphate metabolism related processes (Supporting Data 8), which may be related to the species’ need for higher rates of DNA replication linked to its faster development and higher metabolic rates (Kulkarni *et al.* 2017).

**Table 3:** gProfiler enriched Gene Ontology (Biological Processes only) terms and Reactome pathways for *P. cultripes* and *S. couchii*. Terms and pathways match highlighted annotations in Figure 3

Domain	Term/Pathway	Term/Pathway description	Species	adj. p value
GO: BP	GO:0000375	RNA splicing, via transesterification reactions	*P. cultripes*	<0.001
GO: BP	GO:0000377	RNA splicing, via transesterification reactions with bulged adenosine as nucleophile	*P. cultripes*	<0.001
GO: BP	GO:0000398	mRNA splicing, via spliceosome	*P. cultripes*	<0.001
GO: BP	GO:0001732	Formation of cytoplasmic translation initiation complex	*P. cultripes; S. couchii*	<0.001;<0.001
GO: BP	GO:0002181	Cytoplasmic translation	*P. cultripes; S. couchii*	<0.001;<0.001
GO: BP	GO:0002183	cytoplasmictranslationalinitiation	*P. cultripes; S. couchii*	<0.001;<0.001
GO: BP	GO:0006099	tricarboxylic acid cycle	*P. cultripes*	<0.001
GO: BP	GO:0006119	oxidative phosphorylation	*P. cultripes; S. couchii*	<0.001;<0.001
GO: BP	GO:0006123	mitochondrial electron transport, cytochrome c to oxygen	*S. couchii*	<0.001
GO: BP	GO:0006413	translational initiation	*P. cultripes*	<0.001
GO: BP	GO:0006414	translational elongation	*P. cultripes*	<0.001
GO: BP	GO:0006754	ATP biosynthetic process	*P. cultripes; S. couchii*	<0.001;<0.001
GO: BP	GO:0008380	RNA splicing	*P. cultripes*	<0.001
GO: BP	GO:0009060	aerobic respiration	*P. cultripes*	<0.001
GO: BP	GO:0009141	nucleoside triphosphate metabolic process	*S. couchii*	<0.001
GO: BP	GO:0009142	nucleoside triphosphate biosynthetic process	*S. couchii*	<0.001
GO: BP	GO:0009144	purine nucleoside triphosphate metabolic process	*S. couchii*	<0.001
GO: BP	GO:0009145	purine nucleoside triphosphate biosynthetic process	*S. couchii*	<0.001
GO: BP	GO:0009199	ribonucleoside triphosphate metabolic process	*S. couchii*	<0.001
GO: BP	GO:0009201	ribonucleoside triphosphate biosynthetic process	*S. couchii*	<0.001
GO: BP	GO:0009205	purine ribonucleoside triphosphate metabolic process	*S. couchii*	<0.001
GO: BP	GO:0009206	purine ribonucleoside triphosphate biosynthetic process	*S. couchii*	<0.001
GO: BP	GO:0010927	cellular component assembly involved in morphogenesis	*P. cultripes*	<0.001
GO: BP	GO:0015980	energy derivation by oxidation of organic compounds	*P. cultripes*	<0.001
GO: BP	GO:0015985	energy coupled proton transport, down electrochemical gradient	*P. cultripes; S. couchii*	<0.001;<0.001
GO: BP	GO:0015986	ATP synthesis coupled proton transport	*P. cultripes; S. couchii*	<0.001;<0.001
GO: BP	GO:0019646	aerobic electron transport chain	*S. couchii*	<0.001
GO: BP	GO:0022618	ribonucleoprotein complex assembly	*P. cultripes*	<0.001
GO: BP	GO:0022900	electron transport chain	*P. cultripes; S. couchii*	<0.001;<0.001
GO: BP	GO:0022904	respiratory electron transport chain	*P. cultripes; S. couchii*	<0.001;<0.001
GO: BP	GO:0030239	myofibril assembly	*P. cultripes*	<0.001
GO: BP	GO:0042773	ATP synthesis coupled electron transport	*P. cultripes; S. couchii*	<0.001;<0.001
GO: BP	GO:0042775	mitochondrial ATP synthesis coupled electron transport	*P. cultripes; S. couchii*	<0.001;<0.001
GO: BP	GO:0045214	sarcomere organization	*P. cultripes*	<0.001
GO: BP	GO:0045333	cellular respiration	*P. cultripes*	<0.001
GO: BP	GO:0071826	ribonucleoprotein complex subunit organization	*P. cultripes*	<0.001
GO: BP	GO:0099132	ATP hydrolysis coupled cation transmembrane transport	*P. cultripes*	<0.001
GO: BP	GO:0009063	cellular amino acid catabolic process	*P. cultripes; S. couchii*	0.001;<0.001
GO: BP	GO:0022613	ribonucleoprotein complex biogenesis	*P. cultripes*	0.001
GO: BP	GO:0070972	protein localization to endoplasmic reticulum	*S. couchii*	0.001
GO: BP	GO:0045047	protein targeting to ER	*S. couchii*	0.003
GO: BP	GO:0072599	establishment of protein localization to endoplasmic reticulum	*S. couchii*	0.003
GO: BP	GO:0006122	mitochondrial electron transport, ubiquinol to cytochrome c	*P. cultripes*	0.004
GO: BP	GO:0031032	actomyosin structure organization	*P. cultripes*	0.004
GO: BP	GO:0072376	protein activation cascade	*P. cultripes; S. couchii*	0.004;0.004
GO: BP	GO:0072378	blood coagulation, fibrin clot formation	*P. cultripes; S. couchii*	0.004;<0.001
GO: BP	GO:0010248	establishment or maintenance of transmembrane electrochemical gradient	*P. cultripes*	0.005
GO: BP	GO:0072350	tricarboxylic acid metabolic process	*S. couchii*	0.005
GO: BP	GO:1901605	alpha-amino acid metabolic process	*P. cultripes*	0.006
GO: BP	GO:1901606	alpha-amino acid catabolic process	*P. cultripes*	0.006
GO: BP	GO:0006613	cotranslational protein targeting to membrane	*S. couchii*	0.016
GO: BP	GO:0006614	SRP-dependent cotranslational protein targeting to membrane	*S. couchii*	0.016
GO: BP	GO:0051258	protein polymerization	*P. cultripes*	0.016
GO: BP	GO:0009620	response to fungus	*P. cultripes; S. couchii*	0.018;0.014
GO: BP	GO:0046500	S-adenosylmethionine metabolic process	*S. couchii*	0.018
GO: BP	GO:0032271	regulation of protein polymerization	*P. cultripes*	0.019
GO: BP	GO:0055002	striated muscle cell development	*P. cultripes*	0.021
GO: BP	GO:0016054	organic acid catabolic process	*P. cultripes*	0.025
GO: BP	GO:0046395	carboxylic acid catabolic process	*P. cultripes*	0.025
GO: BP	GO:0034314	Arp2/3 complex-mediated actin nucleation	*P. cultripes*	0.028
GO: BP	GO:0015988	energy coupled proton transmembrane transport, against electrochemical gradient	*P. cultripes*	0.029
GO: BP	GO:0035384	thioester biosynthetic process	*P. cultripes*	0.029
GO: BP	GO:0071616	acyl-CoA biosynthetic process	*P. cultripes*	0.029
GO: BP	GO:0000387	spliceosomal snRNP assembly	*S. couchii*	0.031
GO: BP	GO:0044282	small molecule catabolic process	*P. cultripes*	0.036
GO: BP	GO:0001878	response to yeast	*P. cultripes; S. couchii*	0.038;0.028
GO: BP	GO:0015991	ATP hydrolysis coupled proton transport	*P. cultripes*	0.04
GO: BP	GO:0090662	ATP hydrolysis coupled transmembrane transport	*P. cultripes*	0.04
GO: BP	GO:0099131	ATP hydrolysis coupled ion transmembrane transport	*P. cultripes*	0.04
Reactome	REAC:R-XTR-156842	Eukaryotic Translation Elongation	*P. cultripes*	<0.001
Reactome	REAC:R-XTR-163210	Formation of ATP by chemiosmotic coupling	*S. couchii*	<0.001
Reactome	REAC:R-XTR-71403	Citric acid cycle (TCA cycle)	*S. couchii*	<0.001
Reactome	REAC:R-XTR-8949613	Cristae formation	*S. couchii*	<0.001
Reactome	REAC:R-XTR-390471	Association of TriC/CCT with target proteins during biosynthesis	*P. cultripes*	0.002
Reactome	REAC:R-XTR-3371511	HSF1 activation	*S. couchii*	0.009
Reactome	REAC:R-XTR-159740	Gamma-carboxylation of protein precursors	*P. cultripes*	0.03
Reactome	REAC:R-XTR-196025	Formation of annular gap junctions	*S. couchii*	0.047
Reactome	REAC:R-XTR-5625970	RHO GTPases activate KTN1	*S. couchii*	0.049

**Figure 4:**
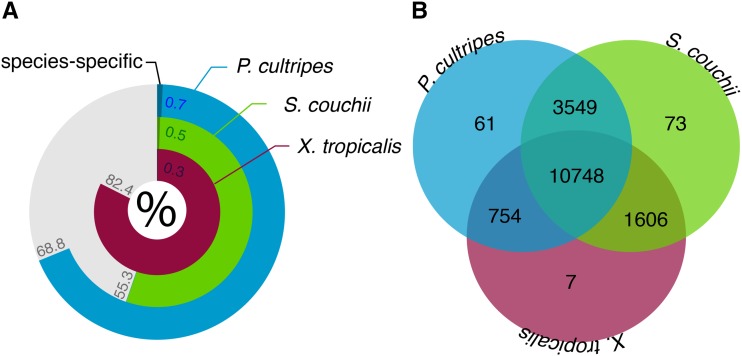
OrthoFinder results showing a) the percentage of genes that could be assigned to orthogroups per species (darker shading represents percentage of genes in species-specific orthogroups) and b) the number of species-specific and shared orthogroups recovered.

OrthoFinder assigned 81,833 genes (64.8% of total) to 16,798 orthogroups. 82.4% of *X. tropicalis* genes could be assigned to orthogroups, compared to 68.8% of *P. cultripes* genes and 55.3% of *S. couchii* genes (Figure 4a). Of the assigned genes, only small fractions of genes were in species-specific orthogroups (*X. tropicalis*: 0.3%, *P. cultripes*: 0.7%, *S. couchii* 0.5%; Figure 3a). Fifty percent (G50) of all genes were in orthogroups with 4 or more genes and were contained in the largest 9,842 orthogroups (O50). There were 10,748 orthogroups (the largest share) with all species present (Figure 4b) and 2,123 of these consisted entirely of single-copy genes. *Pelobates cultripes* and *S. couchii* shared more orthogroups than either did with *X. tropicalis* with 61 and 73 orthogroups being unique to each species respectively, compared to only 7 being unique to *X. tropicalis*.

## Conclusion

*De novo* transcriptome assemblies of the larval phase of two amphibians with vastly differing environmental sensitivity in developmental rate are presented and annotated. Despite having drastically different sized genomes (with that of *Pelobates cultripes* being 2.6 times larger than that of *Scaphiopus couchii*; Liedtke *et al.* 2018), the assemblies are of similar sizes (0.58Gbp *vs.* 0.64Gbp). The assemblies are of high quality, with 83–90% of raw reads mapping onto the transcriptomes, and both transcriptome assemblies consist of >86% full length BUSCO matches with only 2.9% of the assemblies having no corresponding match.

The PANTHER and gProfile overrepresentation tests suggest the two transcriptomes (post express-filtering) are largely comparable in terms of their annotation composition and how they differ to the proteome of *X. tropicalis*. Similarly, the largest share of OrthoFinder orthogroups contains all three species and not unexpectedly (due to their closer phylogenetic affinity; Pyron and Wiens 2011), *P. cultripes* and *S. couchii* share more orthogroups with each other than either does with *X. tropicalis*.

Approximately 40% of the unfiltered assemblies were predicted to be protein coding sequences allowing for extensive annotation and here we provide information on SwissProt proteins (and their GO terms), protein family proteins (Pfam; and their GO terms), protein orthologous groups (eggnog), biological pathways (KEGG database), signal peptide cleave sites (SignalP) and transmembrane protein predictions (TMHMM). The herein provided transcriptomes should therefore serve as an important resource for the advancement in the understanding of amphibian larval transcriptomics.
